# Experimental Study on Chloride Ion Diffusion in Concrete Affected by Exposure Conditions

**DOI:** 10.3390/ma15082917

**Published:** 2022-04-16

**Authors:** Fulai Qu, Jinkai Zhang, Guirong Liu, Shunbo Zhao

**Affiliations:** 1 School of Civil Engineering and Communication, North China University of Water Resources and Electric Power, Zhengzhou 450046, China; qfl@ncwu.edu.cn (F.Q.); zjk15225195573@163.com (J.Z.); sbzhao@ncwu.edu.cn (S.Z.); 2 International Joint Research Lab for Eco-Building Materials and Engineering of Henan, Zhengzhou 450045, China; 3 Collaborative Innovation Center for Efficient Utilization of Water Resources, Zhengzhou 450046, China

**Keywords:** erosion condition, chloride ion, dry–wet cycle, circulating sodium chloride solution, diffusion property

## Abstract

The transport mechanism of chloride ions in concrete is relatively complicated since the erosion process is influenced by many factors. To investigate the effect of exposure conditions on the chloride ion diffusion property, three exposure conditions (long-term immersion in static sodium chloride solution, long-term immersion in circulating sodium chloride solution and dry–wet cycles in circulating sodium chloride solution) were considered in chloride ion diffusion experiments. Experimental results indicated that the chloride ion content at a certain depth increased with erosion age. The chloride ions in static sodium chloride solution transported more rapidly than those under dry–wet cycle conditions. Moreover, the chloride ion content of concrete under dry–wet cycles of the circulating sodium chloride solution was slightly higher than that under long-term immersion in the circulating solution. Based on Fick’s second law, empirical equations for the chloride diffusion coefficient and chloride content at the surface of concrete were proposed by fitting experimental data, and the values of correlation coefficients of different exposure conditions were suggested. By comparison with the experiment results, it was verified that the calculation formula had better applicability. This method could be used to predict and analyze the chloride ion content under different exposure conditions.

## 1. Introduction

Chloride-induced corrosion of steel is a major cause of deterioration in reinforced concrete (RC) structures. If RC structures are exposed to chloride, the chloride ions in surrounding environments will diffuse gradually from the surface to the interior of the concrete with the participation of water [1,2]. When the chloride ion concentration at the reinforcement surface reaches a threshold value, the reinforcements lose the protective effect of the passive layer and begin to rust [3,4,5,6,7]. Due to the volume expansion of corrosion products, the concrete cover of RC structures cracks. These cracks in concrete are likely to increase the transport of chloride ions, thus reducing the structural durability [8,9,10]. Therefore, the resistance of concrete to chloride ion penetration is a very important aspect for the durability and reliability of RC structures exposed to a chloride environment.

It is generally believed that corrosion of reinforcement in concrete requires the combined action of oxygen and water. Compared with the full immersion method, dry–wet cycles can accelerate the corrosion of reinforcements in concrete exposed to sodium chloride solution. Hence, dry–wet cycles are commonly used as an acceleration method for steel corrosion according to previous studies [11,12,13]. Correspondingly, the dry–wet cycle approach has also been employed to investigate chloride ion assault on concrete.

Some research [14,15] showed that when the dry–wet cycle conditions (dry–wet cycle time ratio) changed, there were some differences in concrete saturation, thus having an influence on the critical chloride concentration of concrete, which induced the corrosion of the reinforcement. The studies by Liu et al. [16] and Gao et al. [17] showed that a convection zone existed in concrete under the natural tidal environment and the distribution of chloride ions exhibited high randomness at this convection zone. However, the chloride ions in the inner concrete basically conformed to the characteristics of stable diffusion. Hong et al. [18,19] found that the penetration rate of chloride ions within a certain distance from the concrete surface was accelerated by adsorption action under dry–wet cycles. However, chloride ions in total immersion specimens moved faster than those in dry–wet cycle specimens beyond this distance. Huang et al. [20] used the finite difference approach to examine chloride ion transmission mechanisms during drying and wetting cycles. Moreover, the chloride ion transmission model was constructed for distinct zones in concrete considering diffusion and convection actions. Lai et al. [21] investigated the effects of transverse crack width and water–cement ratio on the chloride profile in concrete subjected to alternating dry–wet cycles.

The erosion of chloride ions in concrete was not only related to the nature of concrete itself (such as concrete pore morphology, porosity, aggregate, etc.), but also related to the environment and the exposure time [21,22,23,24]. In the process of penetration, the chloride diffusion coefficient of concrete was found decreased with time due to the hydration of cementitious material in concrete, as well as the chemical bonding and physical adsorption of chloride ions entering the concrete [25,26,27,28]. The RC structures in the splash zone or tidal zone are subjected to long-term coupling effects of water impact, dry–wet cycles, and chloride ion erosion. Most of the previous studies used a single erosion environment to examine chloride attack, such as full immersion or dry–wet cycles. Few studies have comprehensively considered the impact of the erosion regime and water flow on chloride ion erosion.

To simulate the influence of marine water flow on the diffusion of chloride ions in concrete, an experimental study was carried out on the chloride ion erosion of concrete under three exposure conditions (long-term immersion in static solution, long-term immersion in circulating solution and dry–wet cycle in circulating solution). Based on the experimental measurements of chloride ion content, the models of the apparent chloride diffusion coefficient and surface chloride content were established, respectively. Finally, the accuracy of the adopted model was validated based on the experimental measurements.

## 2. Experimental Program

### 2.1. Specimen Preparation

**Figure 1:**
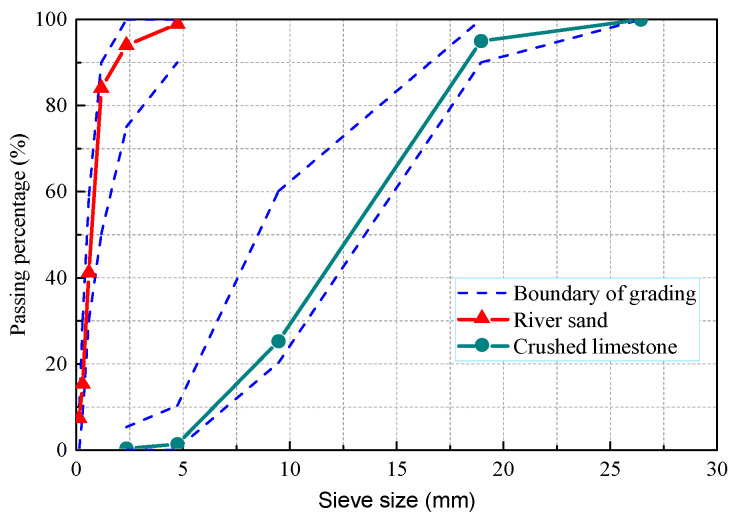
The gradation curves for fine and coarse aggregate.

Ordinary Portland cement 42.5 was used in this study. The chemical compositions and physical properties are given in Table 1. The fine aggregate was natural river sand, with a fineness modulus of 2.71 and an apparent density of 2430 kg/m^3^. Crushed stone was used as a coarse aggregate, with a maximum particle size of 20 mm and an apparent density of 2680 kg/m^3^. The grading curves of the fine aggregate and coarse aggregate are shown in Figure 1. Their performance met the requirements of concrete.

A polycarboxylate-based superplasticizer was incorporated to modify the workability of the concrete. The mixture proportions for concrete were designed according to the Chinese standard GB/T 50081-2019 [29]. The details of the mix properties are shown in Table 2.

Concrete prisms with a size of 150 mm × 150 mm × 300 mm were used for the chloride permeability test to avoid the influence of specimen size. A total of fifteen groups of chloride permeability specimens were prepared, considering three different erosion conditions with five age durations. Moreover, cube specimens with side length of 150 mm were cast to test the compressive strength at the age of 28 days, and the measured compressive strength was 42.6 MPa.

### 2.2. Chloride Ion Erosion Test

After 28 days of curing, the concrete prisms were sealed with wax, with the exception of the chloride erosion surface, to ensure chloride ion penetration in only one-dimensional transport. The ponding test was adopted for determining the chloride content. Specimens for chloride ion permeability were immersed in 10% chloride ion salt solution for three different erosion environments, as shown in Table 3.

**Figure 2:**
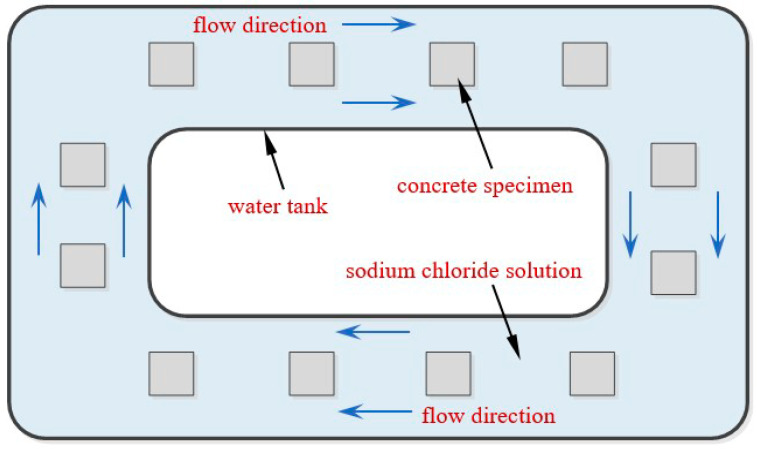
Diagram of erosion setup under flow salt solution.

For the F-DW group, the specimens were placed in a sodium chloride solution tank with 48 h drying and 48 h wetting conditions. The exposure ages were set as 30, 60, 90, 120, and 150 days, respectively. The specimens in the flow chloride solution are shown in Figure 2. A water pump was used to make the sodium chloride solution flow, and the measured flow velocity was 1.2 m/s.

### 2.3. Chloride Ion Content Test

**Figure 3:**
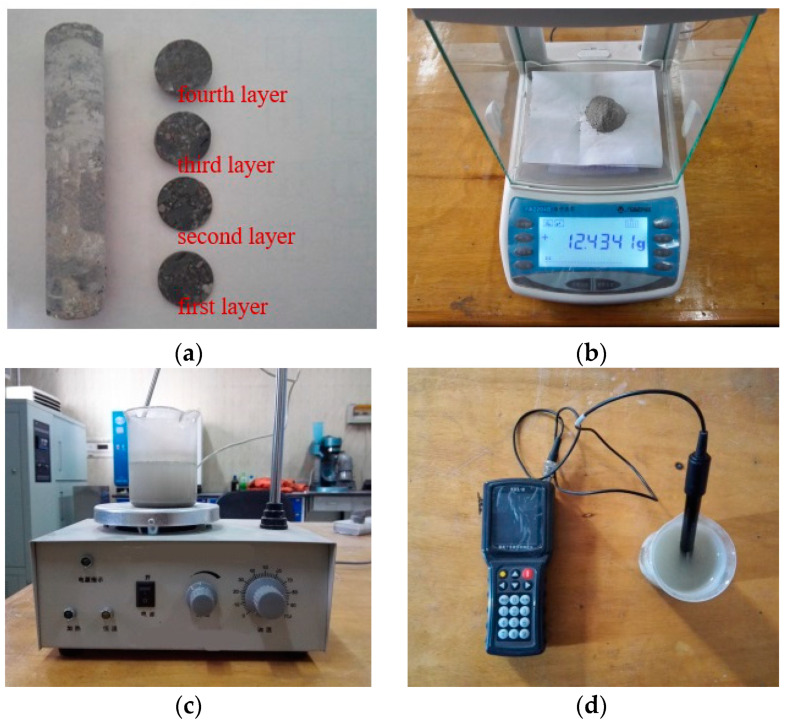
Test progress of chloride ion concentration: (**a**) core drilling and slicing; (**b**) weighing after grinding; (**c**) solution agitation; (**d**) concentration measurement.

After the chloride ion erosion test, the core samples of concrete were drilled from the specimens and then sliced. The slice thickness of each layer from the exposed surface was 2, 3, 5, and 5 mm, respectively. The concrete slices were ground into powder and the coarse particles were discarded. Chloride ion concentration was measured by the rapid chloride measure method in this test. The powder samples were dried and then placed in distilled water to stir. Lastly, the chloride concentrations, which were denoted as the percentage of the binder mass, were measured by a rapid chloride ion detector. The detailed test process is shown in Figure 3.

## 3. Experimental Results and Analysis

### 3.1. Distribution of Chloride Ions

**Figure 4:**
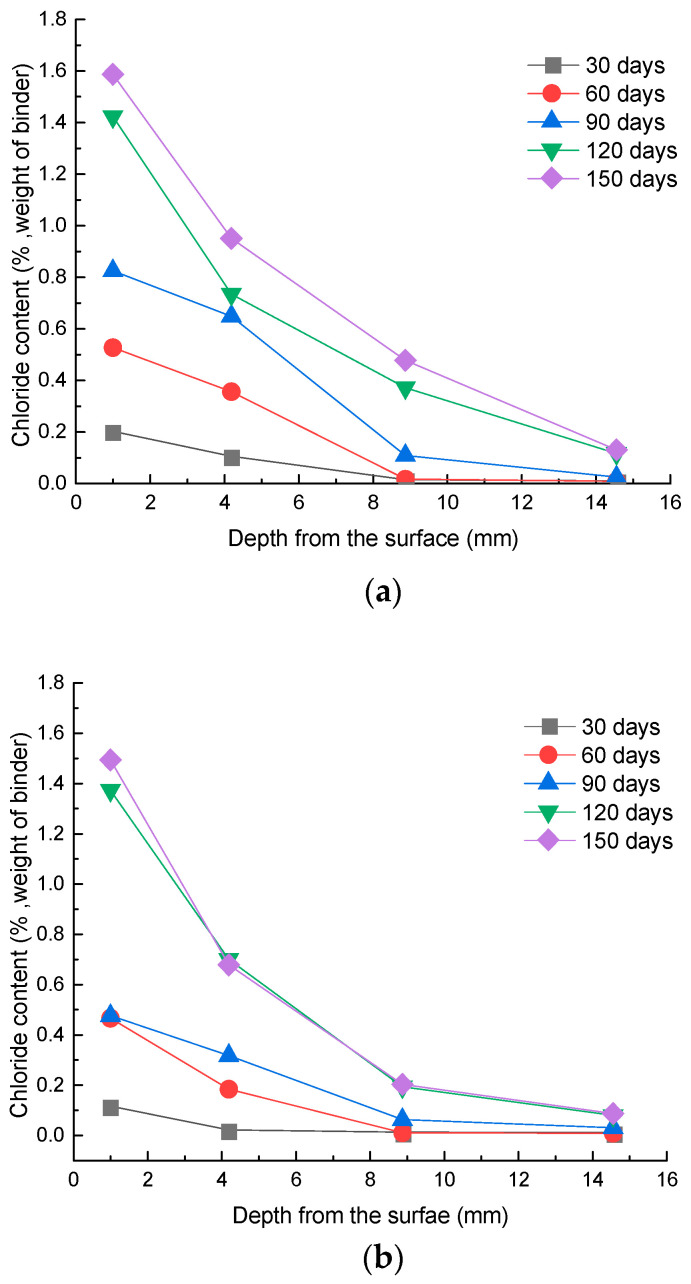

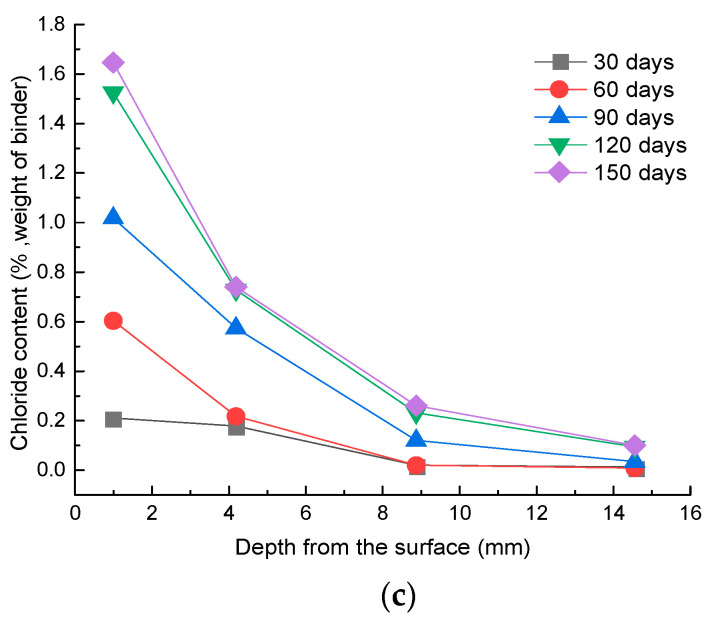
Chloride content at different exposure conditions: (**a**) S-W; (**b**) F-W; (**c**) F-DW.

The distribution of chloride concentration for the concrete specimens under different exposure conditions is exhibited in Figure 4, with the exposure times of 30, 60, 90, 120, and 150 days. It can be seen that under each exposure condition, the chloride ion content decreased with an increase in the concrete depth. Furthermore, the content of chloride ions at the same depth increased with increasing exposure time. The results also indicated that the chloride ion content at a given depth increased rapidly in the early ages. Meanwhile, the chloride ion content increased more slowly after the exposure time of 120 days.

Previous research [18,30,31] has shown that the erosion of chloride ions in surface concrete exposed to the marine environment involves two main transport mechanisms, namely convection and diffusion. For the internal concrete, the penetration of chloride ions in solution was dominated by diffusion. Under the drive of concentration difference, chloride ions migrated from high concentration areas to low concentration areas of the concrete.

**Figure 5:**
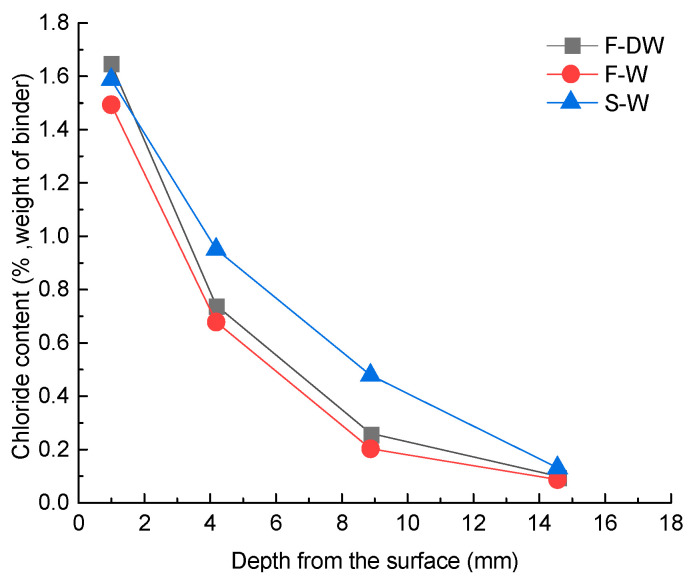
Chloride content after 150 days of exposure.

The profiles of invaded chloride ion content for specimens exposed to distinct corrosion conditions for the age duration of 150 days are shown in Figure 5. It can be seen from the test data that the chloride ion content under the long-term immersion condition of flowing solution (F-W) was lower than that under the long-term immersion condition (S-W), which implied that the flow of the chloride solution did not necessarily accelerate the invasion process of chloride ions in concrete. Moreover, compared with long-term immersion in flowing solution, the concentration of chloride ions was slightly higher in the same layer in concrete under the conditions of dry and wet circulation of flowing solution (F-DW); the results are similar to the findings of Xu’s research [19]. This may be due to the fact that under dry–wet cycles of the flowing solution, the water in the concrete evaporated through the capillary during the drying stage [32,33]. Thus, the humidity inside the concrete was reduced and the negative pressure of the pores enhanced, leading to an increase in the erosion of chloride ions.

### 3.2. Chloride Diffusion Model

The transport of chloride ions in concrete is dominated by diffusion under static solution exposure conditions. Thus, Fick’s second law [34,35,36] is commonly used to describe chloride ion diffusion into concrete, as given by Equation (1).

(1) $$C(x,t)=C_{S}(1-\mathrm{erf}\frac{x}{2\sqrt{D_{a}t}}),\;$$

where *C*(*x*,*t*) is the chloride concentration as a function of depth *x* and time *t*, %; *C*_s_ is the surface chloride content, %; *x* is the depth from the surface; *D*_a_ is the apparent chloride diffusion coefficient in concrete, m^2^/s; *t* presents the exposure duration, s; and erf(·) is the error function,

$$\mathrm{erf}(z)=\frac{2}{\sqrt{\pi }}\int_{0}^{z}\exp(-u^{2})du$$

Due to the existence of a convective zone near the concrete surface under the influence of flowing water, the distribution of chloride in this area did not fully conform to Fick’s second law. The convection zone was relatively small with respect to the thickness of the concrete protective layer, and the chloride content in the convection zone was lower than the value calculated by Fick’s second law. To simplify the analysis of chloride ion transmission in concrete, the diffusion of chloride in the convection and internal zone of concrete was considered to conform to Fick’s second law.

**Figure 6:**
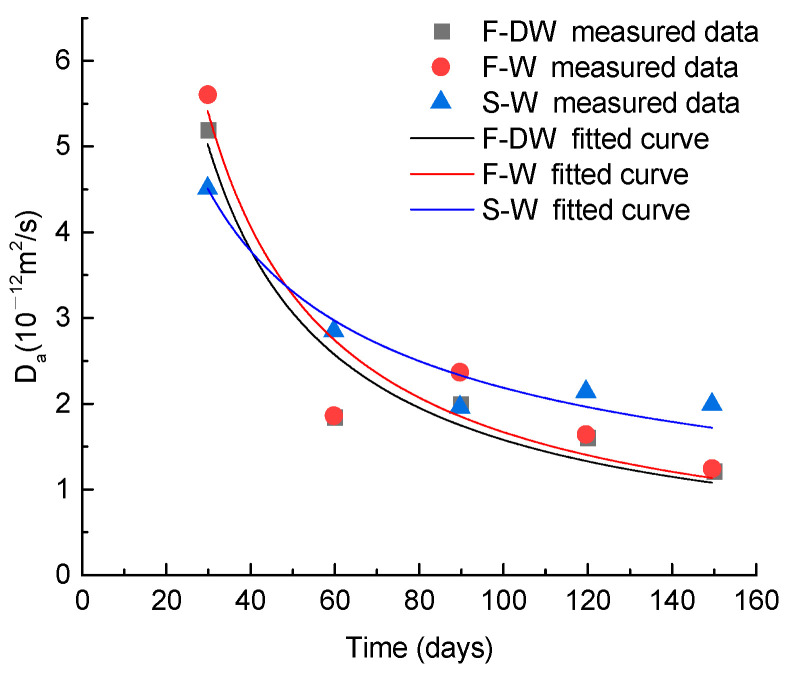
Apparent chloride diffusion coefficient of concrete specimens.

The apparent diffusion coefficient (*D*_a_), as an important parameter in the chloride diffusion model, reflects the relationship between the chloride concentrations at different depths and the surface of the concrete. In this paper, the measured data of chloride concentration at different depths were used to calculate the *D*_a_ value. The measured data and regression results are shown in Figure 6.

According to Figure 6, the value of *D*_a_ decreases with the increase in exposure duration. As the cement hydration reaction proceeds, ettringite continuously forms in the pores of concrete, forming calcium silicate hydrate gels and calcium sulfur aluminate. Therefore, the original, relatively large-diameter or connected pores are gradually filled by the solid-phase products of these cement pastes, and the microstructure tends to be dense [37]. Thus, the diffusion coefficient of chloride ions decreases with time, which is consistent with the findings of existing studies [38]. Moreover, based on previous research [39,40,41,42], the diffusion coefficient of chloride ions is likely to vary with temperature, relative humidity, water flow, and other external factors. The apparent chloride diffusion coefficient *D*_a_ varies for different exposure conditions through the analysis of experimental data. A power function [43,44,45] is adopted to fit the scatter plots of *D*_a_ and exposure time, which can be expressed as follows:

(2) $$D_{a}\left(T\right)=\alpha D_{0}{\left(\frac{T_{0}}{T}\right)}^{m},$$

where *D*_a_(*T*) is the chloride diffusion coefficient at exposure time of *T*, m^2^/s; α, m are influence coefficients associated with the exposure conditions, which are shown in Table 4; *D*_0_ is the reference chloride diffusion coefficient at *T*_0_ (30 days), m^2^/s.

**Figure 7:**
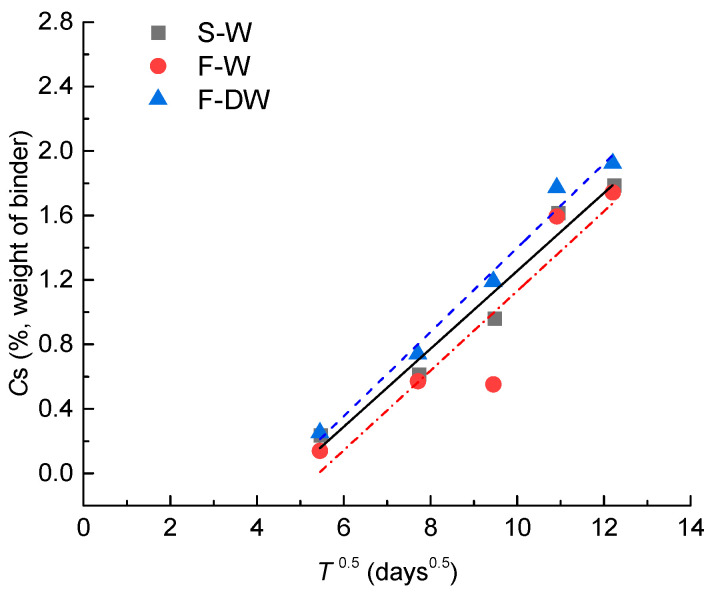
Relationship between surface chloride content and square root of time.

The surface chloride content *C*_s_ in Equation (1) is calculated by chloride diffusion coefficient *D*_0_ and the measured chloride concentration. Based on the data analysis, it is found that the surface chloride content increases with time. The surface chloride content over time can be expressed as

(3) $$C_{s}=k\sqrt{t}-b\geq 0,$$

where k and b are regression coefficients related to the erosion conditions, and the values of k and b could be obtained by fitting the linear function to the scatters, as shown in Figure 7.

### 3.3. Model Validation

The fitting values of apparent chloride diffusion coefficient *D*_a_ and the surface chloride content *C*_s_ can be calculated by Equations (2) and (3), respectively. By substituting them into Equation (1), the chloride content at a given depth and exposure duration time is obtained using the following equation.

(4) $$C(x,t)=(k\sqrt{t}-b)(1-\mathrm{erf}\frac{x}{2\sqrt{D_{a}t}}),$$

**Figure 8:**
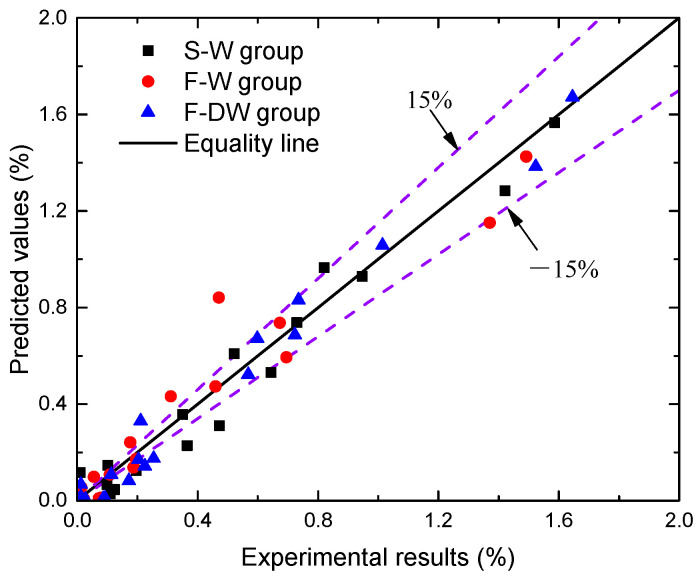
Comparison of the chloride concentrations predicted by the model versus experimental results.

To verify the accuracy of the prediction model in Equation (4), the results of predicted and measured values are plotted in Figure 8. From Figure 8, it can be observed that most of the chloride content values calculated by the model are within a ±15% error margin in comparison to the experimental results, validating the reliability of the prediction model developed in this paper.

**Figure 9:**
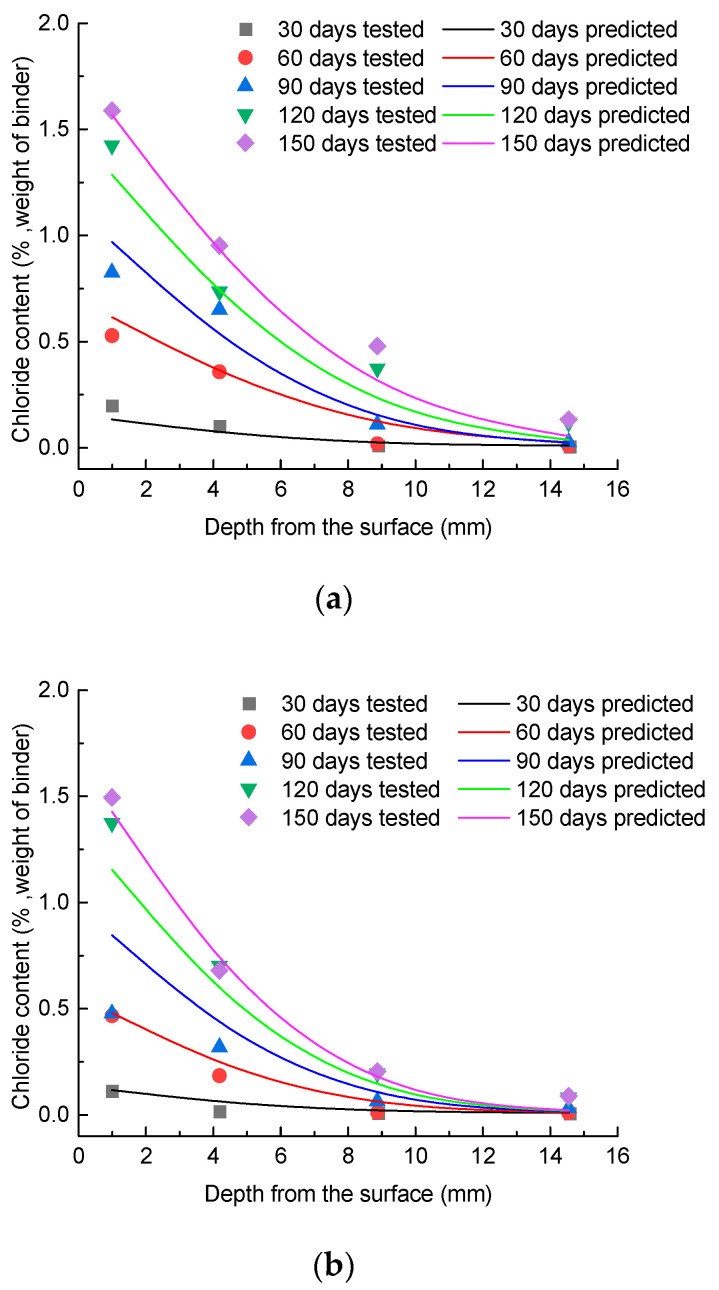

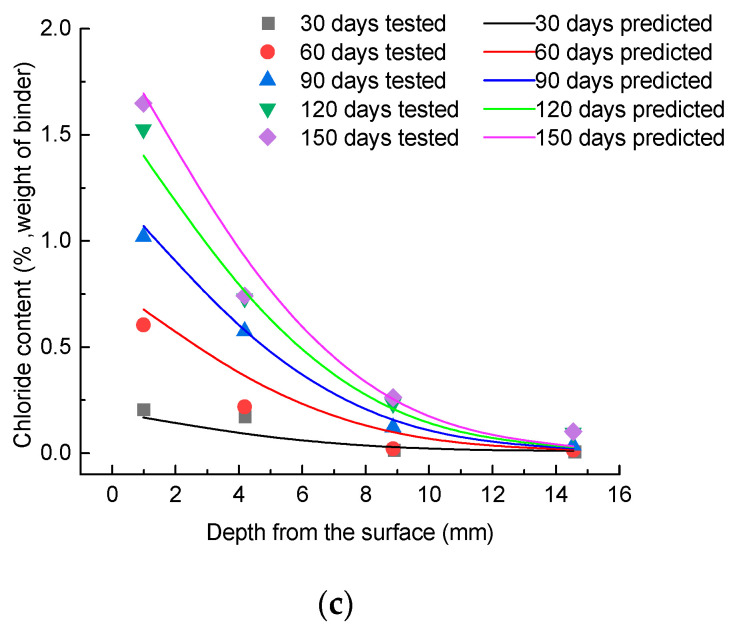
Comparison of predicted and experiment results at different exposure conditions: (**a**) S-W; (**b**) F-W; (**c**) F-DW.

Figure 9 shows the predicted chloride content curves and the experimental data versus exposure duration time *t* (*t* = 30, 60, 90, 120, and 150 days) under different exposure conditions. It can be seen that the magnitude of the chloride content determined by the model agrees well with the experimental results, with the exception of some discrete data.

In this paper, the effects of dry–wet cycles and water flow on the erosion law of chloride ions were studied through experiments. The experimental results show that the flow of the sodium chloride solution does not necessarily accelerate the diffusion of chloride ions into concrete. Moreover, the accelerating effect of dry–wet cycles on the diffusion of chloride in concrete is not obvious. This result is different from the severe corrosion of reinforcements in concrete under dry–wet cycles. The main reason may be that the corrosion of reinforcements in concrete is not only related to the chloride content, but also requires the combined action of oxygen and water. Although the chloride content is not necessarily the largest under dry–wet cycle conditions, the corrosion rate of reinforcements would be accelerated by considering the combined effects of oxygen and water.

The transport mechanism of chloride ions in concrete is complicated because the chloride ion concentration is affected by many factors. Further studies should be carried out on chloride ion erosion in concrete as affected by multiple factors.

## 4. Conclusions

The influence of exposure conditions on chloride transmission in concrete was experimentally investigated in this study. The chloride content in concrete was tested at various depths for different exposure durations. Then, these experimental results were used to investigate the variation rules of the apparent chloride diffusion coefficient and the surface chloride content. Finally, based on Fick’s second diffusion law, a model affected by exposure conditions was proposed to evaluate the chloride diffusion in concrete. The major conclusions of this study are summarized as follows.

The chloride content in concrete varied under different exposure conditions. Under the static solution condition, the chloride content of concrete was generally higher than that under the flowing solution condition. Moreover, the chloride content of concrete was slightly higher in dry–wet cycles of the flowing sodium solution than that in the long-term immersion of the flowing sodium solution. Moreover, the distribution of chloride content in concrete basically conformed to Fick’s second diffusion law, with the exception of the convection zone. The assumed surface chloride content values were calculated by the fitting method of the measured chloride content in concrete. Lastly, in terms of the chloride diffusion coefficient and chloride content at the concrete surface, a model was developed to simulate the chloride content in the concrete under various exposure situations. Furthermore, the validity of this model was proven by comparing the calculated and experimental results.

## Figures and Tables

**Table 1:** Cement chemical composition and physical properties.

CaO (%)	SiO_2_ (%)	MgO (%)	SO_3_ (%)	Al_2_O_3_ (%)	Fe_2_O_3_ (%)	Density (g/cm^3^)	Surface Area (m^2^/kg)	Loss (%)
60.41	21.65	3.46	2.24	4.67	2.98	3.15	337	2.74

**Table 2:** Mixture properties of concrete (kg/m^3^).

Concrete Grade	Cement	Fine Aggregate	Coarse Aggregate	Water	Superplasticizer
C40	478	1172	632	167	6.8

**Table 3:** Exposure conditions of chloride permeability test.

Group No.	State of Solution	Erosion Progress
S-W	static	long-term immersion
F-W	flowing	long-term immersion
F-DW	flowing	dry–wet cycles

**Table 4:** Fitting results of parameters for chloride diffusion coefficient.

Exposure Condition	*D*_0_ (10^−12^ m^2^/s)	α	m	R^2^
S-W	4.50	0.98	0.59	0.926
F-W	5.60	0.97	0.98	0.876
F-DW	5.19	0.97	0.97	0.906

## Data Availability

Not applicable.
